# Impact of smoking on the immunological profile of patients with laryngeal carcinoma

**Published:** 2009-04-25

**Authors:** L Melinceanu, C Sarafoleanu, L Lerescu, C ţucureanu, I Caraş, A Sălăgeanu

**Affiliations:** * ‘SfȦnta Maria’ ENT–CCF Clinic, BucharestRomania; ** #x2018;Cantacuzino#x2019; INCDMI– The Antiinfectious Immunity Laboratory, BucharestRomania

## Abstract

Immunity plays an important role in the prognosis and the natural development of cancer. Previous studies have shown that the presence of tumor in the body could modify the immune response leading to immunosuppression. The aim of this study was to evaluate the immunological changes of patients with larynx squamous cell carcinoma undergoing potentially curative surgery. We assessed the serum levels of cytokines (IL–1, IL–2, IL–4, IL–6, IL–8, IL–10, IFN–gamma, TNF–alpha, GM–CSF), chemokines (MCP–1 and MIP–1alpha) and growth factors (VEGF and bFGF) in laryngeal cancer patients before, during and after surgery. We have used a novel multianalyte XMap profiling technology that allows simultaneous measurement of multiple parameters in small volumes of samples. To investigate the changes in immune mediators ' profile induced by tumor resection, we assessed the culture supernatants of the peripheral blood mononuclear cells (PBMC) derived from the patients, before and after surgery. The results suggested a predominance of a Th2 type of immune response associated with the presence of the tumor (especially in the case of heavy smokers who smoke more than 40 pack–years). However, shifts towards a Th1 type of immune response as well as an improvement of monocyte functions were noticed after surgery.

## Introduction

Larynx cancer is an aggressive disease accompanied, among others, by changes in the mediators involved in the immune response, inflammation and angiogenesis. A better understanding of these disorders can contribute to an early diagnosis, improved prognosis and some new therapeutic opportunities. There were approximately 12,250 new cases of larynx cancer diagnosed in 2008 in the U.S., 9680 men and 2570 women [1,2]. This type of cancer is now frequently diagnosed in younger patients (40–50 years old) and despite the improved diagnosis and treatment, the mortality rate of larynx cancer has not changed significantly (5 year–survival rate of 64%) [1,4]. Among the causes of the low survival rate are the lack of screening methods (clinical examination remains the single solution to early detection) and the late presentation of patients to the specialist as a result of little worrying symptoms (hoarseness) [5]. Tromp and collaborators have shown that the lack of suspicion of cancer at the first consultation is associated with delay in the second consultation among patients who were not diagnosed with cancer initially [6].

Smoking, alcohol and malnutrition are the main risk factors in laryngeal cancer. The risk increases with the number of smoked cigarettes and the number of smoking years. The association of smoking with alcohol consumption increases the risk of laryngeal cancer occurrence by approximately 30 times [1], alcohol favoring carcinogens penetration through mucosa by vasodilatation. Quitting smoking and reducing alcohol consumption are the most effective methods for primary prevention.

### Cytokines in the squamous cell carcinoma of the larynx

The relationship between immunity and cancer is bivalent. A decrease in immunity may influence cancer occurrence and tumor causes a decrease in immunity, thus creating a vicious circle. Molecular mechanisms involved in immunosuppression and mechanisms, by which tumors avoid immune response of the host, remain mostly unknown. Cytokines are glycoproteins that act as mediators of immunity. It was shown that the evolution of the laryngeal squamous cell carcinoma is associated with changes in the levels of several cytokine such as IL–4, IL–6, IL–8,IL–10, GM–CSF (granulocyte macrophage colonyZ–stimulating factor), VEGF (vascular endothelial growth factor), bFGF (basic fibroblast growth factor) [ 7,11].

It was also suggested that high levels of immunosuppressive cytokines are related to the mechanisms used by laryngeal squamous cell carcinoma in order to avoid destruction by the immune system. Cytokines involved in squamous cell carcinoma of the larynx and their functions are presented in Table 1

**Table 1:** Squamous cell carcinoma of the larynx – relevant cytokines and their cellular functions HGF: Hepatocyte growth factor, MIF: Macrophage migration inhibitory factor, PDGF: Platelet–derived growth factor, PG: prostaglandin, TGF–beta:Transforming growth factor, VEGF:Vascular endothelial growth factor, bFGF basic fibroblast growth factor, GM–CSF: granulocyte macrophage colony stimulating factor.

Cytokines	Cellular functions
IL–1	Cytokine secretion
IL–4	Immune suppresion
IL–6	Inflammation regulation, antiapoptosis
IL–8	Angiogenesis
IL–10	Immune suppresion
HGF	Angiogenesis
MIF	Growth regulation
PDGF	Angiogenesis
PGE2	Immune suppresion
TGF–beta	Immune suppresion
VEGF	Angiogenesis, metastasis, chemoattraction
bFGF	Angiogenesis, metastasis
GM–CSF	CD34 mobilisation, immune supression

### Angiogenesis factors in the squamous cell carcinoma of the larynx

Angiogenesis, defined as the sprouting of new vessels from pre–existing ones, is concededly one of the key–steps in tumor growth and progression. Angiogenesis is initiated by paracrine release of specific growth factors such as VEGF, bFGF, PDGF, GM–CSF and G–CSF [12,13]. It has been shown that VEGF is crucial in sustaining, but not initiating, angiogenesis by malignant squamous cells, and that other angiogenic factor(s) mediates early–stage tumor angiogenesis. VEGF was associated with the tumor progression and the aggressive tumor phenotype [14]. Tumors secreting simultaneously three or more growth factors indicate a worse patient prognosis [12].

A new identified growth factor is MIF (macrophage migration inhibitory factor). Suzuki and collaborators have shown that patients with MIF– negative tumors have a worse prognosis than those with MIF–positive tumors, the last responding much better to chemotherapy (carboplatin) [15].

### Th1/Th2 cytokines profile in larynx cancer

There are two major types of immune response associated with the production of distinct sets of cytokines. Cellular immune response is associated with preferential involvement of Th1 phenotype T helper lymphocytes (Th) and production of IFN–gamma, IL–2 and IL–12. Preferential involvement of Th2 phenotype is associated with the development of humoral immune response, and main Th2 cytokines are IL–4 and IL–10. In healthy individuals, there is a balance between these two types of immune response [16]. Being an effective predominantly cellular anti–tumor immune response (Th1 phenotype), it is considered that a predominant Th2 response may be involved in the tumor progression and/or it is associated to immunosuppression. Studies in literature show that there is a change in immune profile from Th1 to Th2 in patients with laryngeal squamous cell carcinoma, resulting in inhibition of cellular immune response [17, 18].

In this study, we assessed immunological changes in patients with laryngeal squamous cell carcinoma undergoing surgery in curative purpose. For this purpose, the cytokines, chemokines and growth factors patterns were determined in patients' serum before surgery (3–4 days), during the surgical act and at 5–7 days after surgery.

To investigate the changes in immune mediators' profile induced by tumor resection, we assessed the culture supernatants of peripheral blood mononuclear cells (PBMC) derived from the patients, before and after surgery. We used Multiplex xMAP technology, which allows simultaneous determination of several parameters, in a small volume of sample.

## Materials and methods

### Patients

10 patients diagnosed with squamous cell carcinoma of the larynx, undergoing surgery in the ENT–Department, ‘Sf. Maria’ Hospital during September 2007–November 2008 were enrolled in this study. The study was approved by the Ethics Committee of ‘Sf. Maria’ Hospital and inclusion in the study and sampling was done after obtaining written informed consent of subjects. The control group consisted of 10 healthy donors.

### Determination of immune mediators

Cytokines and chemokines were measured both in serum (before surgery, during the surgery and after the surgery) and PBMC (*peripheral blood mononuclear cells*) culture supernatants.

Peripheral blood collected by venous puncture in heparin tubes (100U/mL blood) was immediately diluted 1:1 with culture medium (RPMI_1640_ supplemented with 100U/mL penicillin, 100mg/mL streptomycin, and 1mM glutamine). PBMC cells were isolated using gradient density centrifugation (30min, 2000rpm, 21^o^C, Histopaque–1077, Sigma). After the centrifugation, the mononuclear cell ring was collected and cells were washed three times with medium culture (each followed by centrifugation 10min, 1500, 1200 and 1000rpm, respectively). PBMC were resuspended in complete medium culture (RPMI1640 supplemented with 100U/mL penicillin, 100mg/mL streptomycin, 1mM glutamine and 10% fetal calf serum, SFV), counted, plated at concentration of 1.5 x 10^6^cel/ml (200microL/well) in 96–well plates and incubated in the presence of phytohemmagglutinin (PHA, 5 microg/ml) or *E.coli* lipopolysaccharide (LPS, 1microg/mL) for 24 h at 37^o^C in the presence of 5% CO_2_. Culture wells stimulated with phosphate buffered saline (PBS) were used as negative controls. Culture supernatants were collected after 24h of centrifuged incubation (5 min, 1200 rpm) and tested on Luminex 100 platform. The following mediators were tested in sera: TNF–alpha, IL– 6, IL–10, MIP–1alpha (*macrophage inflammatory protein*), VEGF (*vascular endothelial growth factor*), bFGF (*basic fibroblast growth factor*), MCP–1 (*monocyte chemoattractant protein*) (using Human Multianalyte Profiling Base Kit A, RD Systems). The following parameters were tested in PBMC culture supernatants: IFN–gamma, IL–1beta, IL–1 Ra (IL–1 receptor antagonist), IL–2, IL–4, IL–10, using the Milliplex MAP kit (Millipore). Plate reading was performed on the Luminex 100 platform and data were processed with Luminex 100 IS 2.3 Star Station software (separate results in MFI: Mean Fluorescence Intensity and concentrations, pg/mL, for each mediator).

### Statistical analysis

The results are reported as median values, range of variation (25^th^ to 75^th^ percentiles) and standard error of the mean. Statistical analysis was performed by unpaired Mann–Whitney test using SPSS 9.0 software. Statistical significance was defined as a two–sided P value of less than 0.05.

## Results

The demographic and clinical characteristics of patients included in the study are presented in Table 2

**Table 2:** Clinical and demographic characteristics of patients (n=10)

Characteristic	Value
Age, year (average)	54 ± 7.1
Sex:
male	8
female	2
Stage :
T1	1
T2	3
T3	5
T4	1
Stage:
N0	5
N1	2
N2	3
Location:
supraglottic	4
glottic	9
subglottic	3

The patients were clinically evaluated (anamnesis, clinical ENT examination, general physical examination, anesthesiology examination), by laboratory tests, laryngofibroscopy, computed tomography and anatomopathological exam. All patients underwent surgical treatment in ‘Sf. Maria’ ENT Clinical Hospital, consisting of partial or total laryngectomy. Anamneses data, disease onset and development until the time of presentation are presented in Table 3.

**Table 3:** Anamneses data

Smoker Packs – year	Alcohol consumption	Hoarseness(months)
45	Yes	5
60	Yes	12
30	Yes	12
20	Yes	24
52,5	Yes	3
70	Yes	24
25	Yes	6
90	No	8
0	No	2
45	No	12

There was a predominance of smokers, 9 of 10 patients, with an average of 45 pack–years (a pack–year is equivalent to smoking 20 cigarettes daily for one year), a minimum of 20 pack–years, and a maximum of 90 pack–years. Seven of the patients declared that they were chronic alcohol consumers. Patients were presented at an average of 10 months after onset of hoarseness, late presentation to the practitioner having the result that a large percentage of patients were in advanced stages of disease.

**Fig 1:**
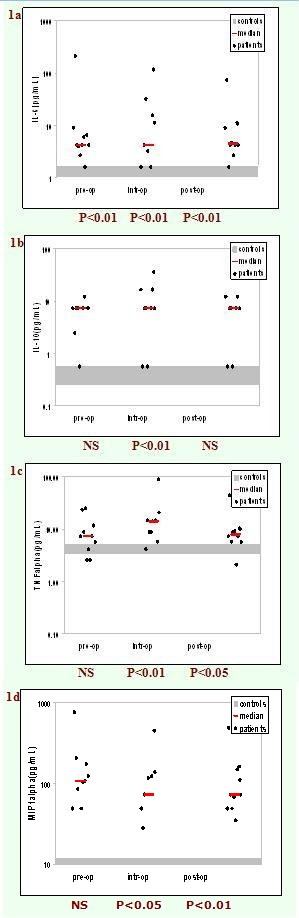
Serum levels of pro-inflammatory cytokines IL–6(a), anti–inflammatory cytokine IL–10(b), pro–inflammatory cytokines TNF–alpha(c) and chemokine MIP–1alpha(d) before, during and after surgery compared to normal values (n=10). Statistically significant compared to normal values by Mann–Whitney test.

**Fig 2:**
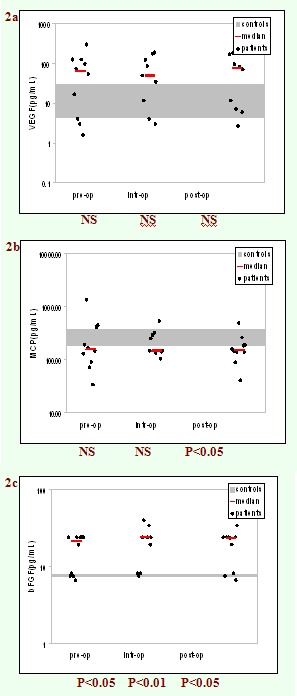
Serum levels of growth factors VEGF(a), MCP 1(b), bFGF(c) before, during and after surgery compared to normal values. Statistically significant compared to normal values by Mann–Whitney test.

The determination of cytokines, chemokines and growth factors was performed using a Milliplex MAP (Millipore) kit on Luminex platform 100 as described in ‘Materials and methods’. The serum level of inflammatory mediators (IL–6, TNFalpha, MCP–1 and MIP–1alpha), anti–inflammatory cytokine (IL–10) and growth factors VEGF and bFGF were measured for the patients at three moments in time, namely before the surgery, during the surgery and after the surgery. The results are presented in Fig 1 and Fig 2.

As it can be seen, the patients had increased levels of IL–6, TNF–alpha, IL–10, MIP–1alpha and bFGF compared to healthy controls, regardless of the time of measuring. The differences reached a statistical significance for IL–6 measured either before or after the surgery (P<0.01 vs. control group). Moreover, the serum concentrations of all the mediators measured during surgery were statistically significantly higher than the control group.

**Fig 3:**
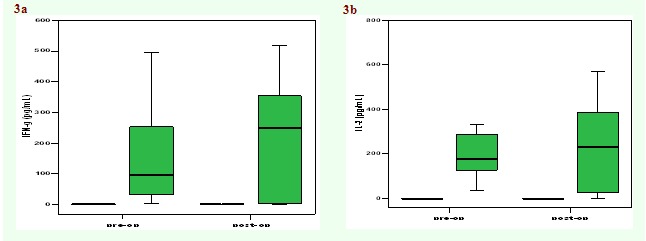
Median values of concentrations of IFN–gamma (a), IL–2 (b) in culture supernatants of PBMC isolated from patients with laryngeal cancer before and after surgery (n = 10).

**Fig 4:**
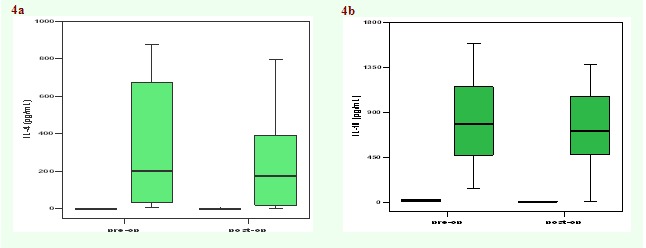
Median values of concentrations of IL–4 (a) and IL–10 (b) in culture supernatants of PBMC isolated from patients with laryngeal cancer before and after surgery (n = 10).

**Fig 5:**
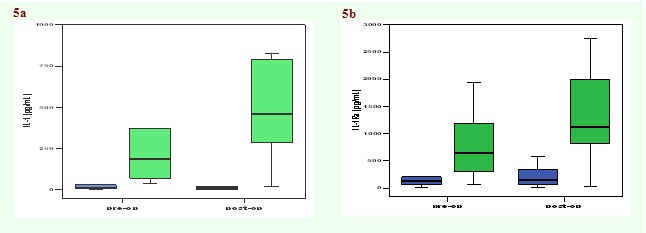
Median values of concentrations of IL–1 (a) and IL–1Ra (b) in culture supernatants of PBMC isolated from patients with laryngeal cancer before and after surgery (n = 10).

Cytokine levels were also measured in PBMC culture supernatants, before and after surgery (Fig 3 and Fig 4). We noticed a trend towards improvement of cellular response (evaluated by the level of IFN–alpha and IL–2 secreted by T lymphocytes stimulated with PHA) after surgery, although the differences were not statistically significant. Moreover, a slight decrease in IL–4 and IL–10 levels (Fig 4) as well as an increase of IL–1 and IL–1 Ra after surgery, was noticed (Fig 5).

**Fig 6:**
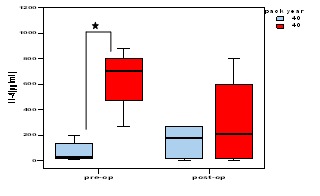
The median concentrations of IL–4 in culture supernatants of PBMC, according to the number of cigarettes smoked, before and after surgery. Statistically significant compared to normal values by Mann–Whitney test.

When analyzing the results in the clinical context we noticed a statistically significant difference between the IL–4 levels in PBMC culture supernatants, in the subgroup of patients who smoked more than 40 pack–years compared to those who smoked less than 40 pack–years, which suggests an influence of smoking on the immune response (Fig 6).

## Discussion

In this study, we examined several immune parameters in patients with squamous cell carcinoma of the larynx, preoperatively and postoperatively, in an attempt to identify the changes induced by the presence of the tumor and/or surgical act. We have used the xMAP novel technology, which proved to be an effective approach to investigate complex networks (such as cytokines network that controls the Th1/Th2 balance) [19].

Our results revealed distinct immunological profiles before, during and after operation. Before surgery, the serum concentration of IL–6 was statistically and significantly higher in cancer patients than in healthy controls (P<0.01, Mann–Whitney test) which, according to the literature data, suggests that the tumor is associated with inflammation [20,21]. An inflammatory response was noticed during the operation, which was characterized by a statistically significant increase in concentration of all tested inflammatory mediators versus control group. Increased concentration of growth factors VEGF and bFGF (statistically significant for bFGF, P <0.05–before and after surgery, and P <0.01–during surgery) could be explained by the involvement of these factors both in the neo–angiogenesis (before surgery) and in the repairing of the tissue (during and after surgery). Surprisingly, serum levels of MCP–1 in cancer patients were lower than in control group (statistically significant, P<0.05, after surgery). The monocyte chemo attractant protein– 1 (MCP–1) has been shown to act as a chemokine in the recruitment of monocyte/macrophages during inflammation states. It acts as an important factor in the cytokine network, which regulates tumor proliferation. However, literature data are controversial regarding the level of MCP–1 in cancer patients. Several studies indicated an increase of the MCP–1 in breast cancer and melanoma [22, 23] and others showed that in patients with gastric cancer, serum level of MCP–1 decreased according to the progression of the disease, probably reflecting its local consumption [24].

In this study, the cytokine production in PBMC of patients with larynx cancer stimulated ex vivo was analyzed before and after the operation. A trend towards an improved cellular immune response (increased amounts of IFN–gamma and IL–2) was noticed after surgery. A slight decrease in the level of IL–4 (a prototype Th2 cytokine) and IL–10 was also noticed after surgery. Although differences were not statistically significant, they might suggest that Th1/Th2 balance shifts towards Th2 dominance in patients after surgery, hypothesis supported by literature data [18,21]. On the other hand, an increase of IL–1 (a pro–inflammatory cytokine) and IL–1Ra anti–inflammatory mediator was noticed postoperatively, which suggests an improvement in monocyte functions. There was no correlation between the level of cytokines determined in the culture supernatant of PBMC and the serum levels of cytokines or the clinical data of patients (stage, degree of differentiation, tumor size or location or number of lymph node metastasis). However, when larynx cancer patients were divided in two subgroups, according to their smoking status, we noticed a statistically significant difference in the level of IL–4 produced by T lymphocytes, i.e. patients who smoked more than 40 pack–years had increased levels of this cytokines (P <0.05, vs. patients who smoked less than 40 pack–years). This is considered an important finding, as increased levels of IL–4 characterize a Th–2 type of immune response. Recent literature data showed that a predominant Th2–type response was induced by components extracted from cigarette smoke through the suppression of dendritic cell function [25].

## Conclusions

This study illustrates different cytokine patterns in serum and PBMC culture supernatants for patients with larynx squamous cell carcinoma undergoing potentially curative surgery. An inflammatory pattern associated with the presence of the tumor was identified. Our results also revealed the inflammatory status associated with surgery and suggested a shift towards a Th1–type of immune response postoperatively. A predominance of the Th2–type of immune response was identified for heavy smokers. Despite the limitation related to the small number of patients investigated, the results provide important information on the immunological changes in patients with laryngeal cancer, after surgery. Further studies are needed to evaluate these changes on a larger number of patients and for a longer period of time after surgery.
